# Supplementary material for: The Relationship between Dietary Patterns and High Blood Glucose among Adults Based on Structural Equation Modelling
Source: Nutrients. 2022 Oct 3;14(19):4111. doi: 10.3390/nu14194111 (PMC9570980; doi:10.3390/nu14194111)
Supplement: Supplementary file 1 [file nutrients-14-04111-s001.zip › nutrients-1929188-supplementary.pdf]

Supplementary Table S1. Food Groupings Used in Factor Analysis.

Food group	Example of food items
Main grains	Rice and wheat and their products
Other grains	Corn, barley, buckwheat
Vegetables	Spinach, canola, carrot, Chinese cabbage, potato, onion
Red meats and its products	Pork, beef, lamb and those products
Eggs	Whole eggs, yolk, preserved eggs
Fruits	Fresh and canned (no added sugar) fruits
Condiments	Sauce, vinegar, salt, preserved vegetables
Soy products	Soybeans, soymilk, tofu
Milk and its products	Whole milk, skim milk, flavored milk, cheese, yogurt
Seafood	Fresh fish, dried fish, shellfish, shrimp
Nuts and seeds	Sesame, sunflower, peanuts, walnuts, hazelnuts, pine-nuts
Beverages	Fruit or flavored drinks, fruit juice, soft drinks
Alcoholic beverages	Beer, rice wine, white wine
Pastry snacks	Cakes, pancake, mooncake
Animal oils	Butter, lard, sheep oil
Poultry	Chicken, duck meat
Vegetable oils	Colza oil, soybean oil, peanut oil, rapeseed oil
Tuber and starches	Tubers and starches
Sugars and preserves	Sugars, confectionery, preserves
Dry legumes	Mung beans, adzuki lentils, kidney beans
Animal viscera	Viscera products of animals
Fungi and algae	Fungus and algae

Supplementary Table S2. Factor Loadings for three Dietary Patterns Derived from Factor Analysis.

Food group	Modern dietary pattern	Fruit-milk dietary pattern
Red meats and its products	0.615	
Vegetables	0.584	
Seafood	0.465	
Condiments	0.430	
Fungi and algae	0.347	0.272
Whole grains	-0.341	0.253
Main grains	0.338	-0.304
Poultry	0.285	
Tuber and starches	-0.258	
Sugars and preserves		
Animal viscera		
Wine		
Milk and its products		0.638
Fruits		0.601
Eggs	0.311	0.474
Nuts and seeds		0.407
Pastry snacks		0.332
Vegetable oils		-0.323
Soy products		
Animal oils		
Dry legumes		
Beverages		
